# Impact of administration timing of subanesthetic doses of esketamine on emergence agitation in patients undergoing nasal endoscopic surgery: a randomized controlled trial

**DOI:** 10.3389/fmed.2026.1978040

**Published:** 2026-10-28

**Authors:** Xiaolan Wen, Wei Sun, Wenhui Chen, Longmei Guo, Yan Zhang, Shutong Lv, Yuxuan Liu, Zeting Qiu

**Affiliations:** 1 Department of Anesthesiology, the First Affiliated Hospital of Shantou University Medical College, Shantou, China; 2 Department of Anesthesiology, the People’s Hospital of Nanhai Economic Development Zone (Foshan Nanhai District Geriatric Hospital), Foshan, China

**Keywords:** emergence agitation, esketamine, nasal endoscopic surgery, subanesthetic dose, timing of administration

## Abstract

**Introduction:**

Emergence agitation (EA) is a common complication following general anesthesia, particularly after nasal endoscopic surgery with nasal packing. This study investigated whether the administration timing of subanesthetic esketamine influences its efficacy in preventing EA.

**Methods:**

In this randomized, double-blind, placebo-controlled trial registered at the China Clinical Trial Registry (No. ChiCTR2500115004), 108 adult patients undergoing elective nasal endoscopic surgery were randomized (1:1:1) to receive intravenous normal saline (Group C), esketamine 0.25 mg.kg⁻^1^ during anesthesia induction (Group E1), or esketamine 0.25 mg.kg⁻^1^ 20 min before surgical completion (Group E2). The primary outcome was the incidence of EA in the post-anesthesia care unit (PACU), defined as a Riker Sedation-Agitation Scale (SAS) score ≥ 5. Secondary outcomes included SAS scores, postoperative NRS pain scores, hemodynamics, and adverse events.

**Results:**

Ninety-five patients completed the study (Group C: *n* = 32; Group E1: *n* = 32; Group E2: *n* = 31). Baseline characteristics were comparable across groups. The overall EA incidence differed significantly among groups (13/32 [40.6%] in C, 8/32 [25.0%] in E1, 3/31 [9.7%] in E2; *P* = 0.018). Group E2 had a significantly lower EA incidence (absolute risk reduction [ARR], 30.9%, [95% CI, 11.0%–50.9%]; number needed to treat [NNT], 3.2 [95% CI, 2.0–9.1]; *P* = 0.011) and lower SAS scores (adjusted *P* = 0.016) than Group C, whereas Group E1 showed no significant difference compared with Group C (*P* = 0.287). Postoperative pain scores, rescue analgesia, extubation time, PACU stay, hemodynamic stability, and adverse event rates were comparable across all groups.

**Conclusions:**

The preventive effect of subanesthetic esketamine on EA is timing-dependent. Administration 20 min before the end of surgery (0.25 mg.kg⁻^1^ IV) significantly reduces EA incidence in nasal endoscopic surgery patients. However, this single-center finding requires multicenter validation, and NRS scales remain limited in capturing packing-related suffocation discomfort.

**Clinical Trial Registration:**

identifier ChiCTR2500115004.

## 1 Introduction

Emergence agitation (EA) is a pathological state characterized by psychomotor agitation, excessive motor activity, and perceptual disturbance occurring during early recovery from general anesthesia (1, 2). Although EA is generally self-limiting and resolves within several minutes to an hour, it can lead to clinically significant adverse events, including patient or staff injury, surgical site bleeding, accidental removal of endotracheal tubes, respiratory depression, and increased healthcare costs (3, 4). The incidence of EA is notably higher in specific surgical populations, particularly in otolaryngologic procedures, where it has been reported to reach up to 55.4% (5, 6).

Following nasal endoscopic surgery, most patients require nasal packing for 48 to 72 h to achieve hemostasis. Consequently, patients are forced to breathe through the mouth, exacerbating discomfort and inducing a sense of asphyxia (7, 8). Postoperative EA in these patients may cause bleeding at the surgical site and blood accumulation in the oral cavity, potentially obstructing the upper airway (9). While most anesthesiologists tend to treat EA reactively with sedatives and opioids, this approach may inadvertently increase the risk of respiratory depression during the emergence phase (10). Therefore, prevention of EA is of greater clinical importance and safety than treatment (11).

Esketamine, the S(+) enantiomer of ketamine, is a non-competitive N-methyl-D-aspartate (NMDA) receptor antagonist with approximately twice the analgesic and anesthetic potency of racemic ketamine (12). At subanesthetic doses (defined as an IV bolus of ≤0.3 mg.kg^−1^), esketamine provides sedation, amnesia, and analgesia without causing significant respiratory depression (13, 14). Its prophylactic efficacy against EA is mediated through multiple mechanisms, including central nervous system stabilization, sympathetic suppression, and anti-inflammatory effects (15, 16).

Recently, the timing of esketamine administration has emerged as a critical variable influencing its efficacy in preventing EA. Pharmacokinetic data indicate that peak plasma concentrations occur within 1 to 5 min, with an elimination half-life of approximately 7 to 12 h (17, 18). Administration during anesthetic induction may effectively blunt the intubation response, but its residual concentration may be insufficient to prevent EA during the extubation phase. Conversely, administration towards the end of surgery may align the drug's peak effect with extubation, optimally suppressing sympathetic hyperreactivity.

Previous studies evaluating ketamine or esketamine for EA prevention have employed varying doses and administration timings. Demir et al. showed that subanesthetic ketamine given 20 min before the end of surgery reduced EA in rhinoplasty patients (19), while Omidvar et al. demonstrated that ketamine 0.5 mg.kg^−1^ or lidocaine 1.5 mg.kg^−1^ IV given 20 min before surgery end was effective in preventing EA and controlling pain (20). However, systematic comparison of the optimal administration timings of subanesthetic esketamine for EA prevention in nasal endoscopic surgery is lacking.

To address this knowledge gap, we designed this randomized, placebo-controlled trial to compare two distinct timings of esketamine administration: during anesthetic induction versus 20 min before the end of surgery. The primary objective was to determine the optimal timing for EA prevention, while the secondary objective was to evaluate its effects on postoperative pain control and recovery profiles in patients undergoing nasal endoscopic surgery under general anesthesia.

## 2 Materials and methods

### 2.1 Study design and ethics

This was a single-center, prospective, randomized, double-blind, placebo-controlled clinical trial conducted between January 1, 2026 to June 30, 2026 at the First Affiliated Hospital of Shantou University Medical College. The study was approved by the institutional ethics committee (Approval No. B-2025-150) and registered at the China Clinical Trial Registry (Registration No. ChiCTR2500115004) prior to patient enrollment. All patients provided written informed consent before enrollment. The study was conducted in accordance with the Declaration of Helsinki and Good Clinical Practice guidelines.

### 2.2 Participants

Patients aged ≥18 years with American Society of Anesthesiologists (ASA) physical status I-II, scheduled for elective nasal endoscopic surgery under general anesthesia with tracheal intubation, were eligible for enrollment. Pre-randomization exclusion criteria were: (1) severe dysfunction of vital organs (heart, liver, kidney); (2) poorly controlled hypertension; (3) known allergy to esketamine; (4) history of drug abuse; (5) history of psychiatric or neurological disorders; (6) untreated or inadequately treated hyperthyroidism. Post-randomization withdrawal criteria were: (1) intraoperative change of anesthesia method; (2) withdrawal of consent by the patient or their family; (3) surgery duration ≤20 min (which structurally precluded the timed end-of-surgery administration); (4) conversion to ambulatory surgery; (5) changes to the planned surgical procedure.

### 2.3 Randomization and blinding

Patients were randomly assigned in a 1:1:1 ratio to three groups using a computer-generated random number sequence. Group allocation was sealed in sequentially numbered opaque envelopes. On the day of surgery, an independent researcher opened the envelope and prepared the study drug. The study drug and placebo were identical in appearance (colorless solutions). All groups received intravenous (IV) injections at two distinct time points (during induction and 20 min before the end of surgery) using either esketamine or normal saline to maintain the blind. The anesthetist administering the drug, the patient, and the outcome assessor (Post-Anesthesia Care Unit [PACU] nurses) were all blinded to group assignment. Unblinding occurred after completion of all data collection and verification.

### 2.4 Study groups and interventions

Group E1 (Induction): Received esketamine 0.25 mg.kg⁻^1^ IV during anesthesia induction and normal saline 20 min before the anticipated end of surgery. Group E2 (End of surgery): Received normal saline during anesthesia induction and esketamine 0.25 mg.kg⁻^1^ IV 20 min before the anticipated end of surgery. Group C (Control): Received normal saline IV at both time points.

### 2.5 Anesthesia protocol

Upon arrival in the operating room, standard monitoring was applied, including pulse oximetry (SpO₂), heart rate (HR), mean arterial pressure (MAP), electrocardiography (ECG), and bispectral index (BIS). After establishing a 22G intravenous line and 3 min of pre-oxygenation, anesthesia was induced. Patients were premedicated with intravenous penehyclidine hydrochloride 0.01 mg.kg⁻^1^. Anesthesia induction was then performed with ciprofol 0.4 mg.kg⁻^1^, sufentanil 0.4 µg.kg⁻^1^, and vecuronium 0.1 mg.kg⁻^1^, concurrently with the first study drug injection (esketamine or saline). After loss of consciousness and confirmation of adequate neuromuscular blockade, tracheal intubation was performed using a 7.0-mm internal diameter tube inserted to 22 ± 2 cm depth. Anesthesia was maintained with propofol 2-4 µg.ml⁻^1^ and remifentanil 3-4 ng.ml⁻^1^ via target-controlled infusion (TCI), with intermittent vecuronium for neuromuscular blockade. Mechanical ventilation was delivered in volume-controlled mode with tidal volume 6-8 ml.kg⁻^1^ and respiratory rate 10-15 breaths.min⁻^1^. The depth of anesthesia was titrated to maintain a BIS value between 40 and 60. Twenty minutes before the anticipated end of surgery, the second study drug injection (esketamine or saline) was administered. All groups received ondansetron 4 mg IV for antiemetic prophylaxis and flurbiprofen axetil 50 mg IV for preventive analgesia. At the end of surgery, sugammadex 2-4 mg.kg⁻^1^ IV was administered to reverse residual neuromuscular blockade. Patients were transferred to the PACU. Tracheal extubation was performed after full consciousness, recovery of protective airway reflexes, and normalization of spontaneous ventilation. Patients were discharged to the ward when Steward recovery score ≥ 4 and vital signs were stable.

### 2.6 Outcomes

The primary outcome was the incidence of emergence agitation (EA) during the PACU stay, defined as a Riker Sedation-Agitation Scale (SAS) score ≥ 5. The SAS score ranges from 1 (unarousable) to 7 (dangerous agitation), with a score of 4 indicating a calm and cooperative status.

The secondary outcomes included: (1) postoperative pain intensity assessed by the Numeric Rating Scale (NRS) at 2 h, 4 h, 6 h, and 24 h postoperatively; (2) the requirement for rescue analgesics in the PACU and ward; (3) intraoperative hemodynamic parameters (HR, MAP, BIS) recorded at eight time points: pre-induction (T0), immediately after intubation (T1), 10 min post-intubation (T2), end of surgery (T3), PACU entry (T4), extubation (T5), 10 min post-extubation (T6), and PACU discharge (T7).

Other recorded variables included: surgery and anesthesia durations, intraoperative medication consumptions (propofol, remifentanil, sufentanil), fluid administration (crystalloid, colloid), estimated blood loss, use of vasoactive drugs, extubation time, PACU stay duration, time to nasal pack removal, and hospital length of stay. Adverse events were prospectively monitored throughout the study period, including nausea, vomiting, dizziness, drowsiness, sore throat, and urinary retention.

### 2.7 Sample size calculation

Sample size was calculated using PASS 2021 software based on previously reported EA incidence rates of 55.4% in nasal surgery without prophylaxis (6), 20% with pre-induction esketamine, and 8.6% with end-of-surgery ketamine (19). With *α*=0.05 (two-sided) and power=0.80, a minimum of 93 patients was required (31 per group) was required for the final analysis. Accounting for an estimated post-randomization dropout rate of 10%, approximately 104 patients were needed. To accommodate a 1:1:1 allocation ratio using a permuted block size of 6, our precise target was set to randomize 108 patients (36 per group). Factoring in an anticipated screen failure rate of 8% to 10%, we expected to screen between 117 and 120 patients. In practice, screening was concluded after exactly 117 patients were assessed, which successfully yielded the pre-specified target of 108 randomized participants.

### 2.8 Statistical analysis

Continuous variables were tested for normality using the Shapiro–Wilk test. Normally distributed data are presented as mean ± standard deviation (SD) and were compared using one-way analysis of variance (ANOVA). Non-normally distributed data are presented as median (interquartile range, IQR) and were compared using the Kruskal–Wallis test. Categorical variables are presented as frequencies (%) and were compared using the chi-squared test or Fisher's exact test as appropriate. EA incidence was compared using the chi-squared test, with *post-hoc* unadjusted pairwise comparisons using chi-squared tests with Bonferroni correction (adjusted *α*’ = 0.05/3 = 0.0167), which served as the primary confirmatory analysis. For the primary outcome, absolute risk reduction (ARR) and number needed to treat (NNT), along with their corresponding 95% confidence intervals (CIs), were calculated using the Wald method. SAS scores were compared using the Kruskal–Wallis test with Bonferroni-corrected Mann–Whitney U tests for pairwise comparisons. Multivariable logistic regression was performed as an exploratory secondary analysis to identify independent risk factors for EA, with group assignment, age, sex, BMI, ASA physical status, duration of surgery, and intraoperative blood loss included as covariates, where results are presented as adjusted odds ratios (ORs) with 95% confidence intervals (CIs). Subgroup analyses stratified by sex and median of age were performed, with treatment-by-subgroup interaction assessed using logistic regression models. Repeated hemodynamic measurements (HR, MAP, BIS) were analyzed using two-way mixed ANOVA (between-subject factor: group; within-subject factor: time), with Greenhouse-Geisser correction for violations of sphericity. No multiplicity adjustments were applied to secondary outcomes. Consequently, nominally significant *P*-values among secondary analyses should be considered exploratory and hypothesis-generating rather than confirmatory. For this trial, the primary analysis was conducted on a per-protocol (PP) basis, defined as all randomized patients who completed the assigned intervention without protocol violations. Additionally, a sensitivity analysis was performed on the Full Analysis Set based on the modified intention-to-treat (mITT) principle. The FAS population included all randomized patients who received at least one study drug injection. A two-sided *P*-value < 0.05 was considered statistically significant. All analyses were performed using Python version 3.13 (utilizing scipy, statsmodels, and pingouin libraries).

## 3 Results

### 3.1 Patient characteristics

A total of 117 patients were screened for eligibility. Based on pre-specified criteria, 108 were randomized. After post-randomization exclusions and dropouts, 95 patients were included in the final analysis: Group C (*n* = 32), Group E1 (*n* = 32), and Group E2 (*n* = 31) (Figure 1). Baseline demographic and clinical characteristics were comparable across the three groups (Table 1). There were no significant differences in age, sex distribution, BMI, ASA classification, or medical history.

**Figure 1:**
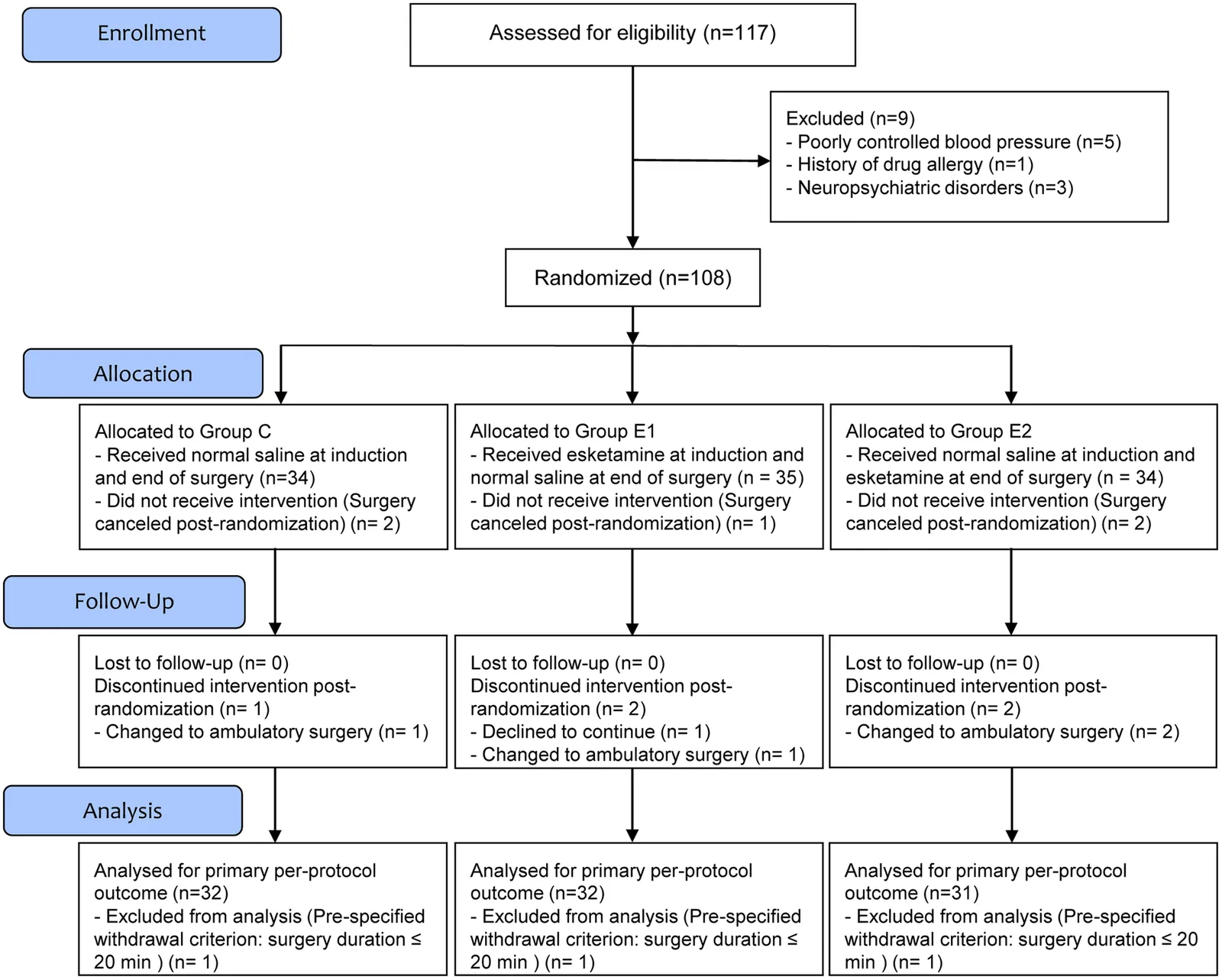
CONSORT flow diagram of patient enrollment, allocation, follow-up, and analysis. The primary analysis was conducted on a per-protocol basis (*n* = 95), including all randomized patients who completed the study without major protocol deviations or pre-specified withdrawal triggers (surgical duration ≤20 min). CONSORT, Consolidated Standards of Reporting Trials; Group C, control group (received normal saline); Group E1, received esketamine during anesthesia induction; Group E2, received esketamine 20 min before the anticipated end of surgery; n, number of patients.

**Table 1:** Baseline demographic and clinical characteristics of patients.

Characteristic	Group C (*n* = 32)	Group E1 (*n* = 32)	Group E2 (*n* = 31)	*P* Value
Sex, n (%)				0.406
Male	23 (71.9)	21 (65.6)	25 (80.6)	
Female	9 (28.1)	11 (34.4)	6 (19.4)	
Age (years)	43.53 ± 15.02	40.12 ± 13.71	43.48 ± 14.67	0.562
Height (cm)	166.16 ± 8.02	167.75 ± 6.27	168.58 ± 7.78	0.419
Weight (kg)	64.81 ± 10.26	65.42 ± 12.85	65.47 ± 11.31	0.968
BMI (kg.m⁻^2^)	23.48 ± 3.37	23.10 ± 3.43	23.02 ± 3.49	0.850
ASA status, n (%)				0.898
Class I	14 (43.8)	14 (43.8)	12 (38.7)	
Class II	18 (56.2)	18 (56.2)	19 (61.3)	
Medical history, n (%)				
Hypertension	1 (3.1)	0 (0.0)	0 (0.0)	>0.999
Diabetes mellitus	0 (0.0)	1 (3.1)	0 (0.0)	>0.999
Hepatitis B virus infection	1 (3.1)	1 (3.1)	0 (0.0)	>0.999
Bronchial asthma	1 (3.1)	0 (0.0)	0 (0.0)	>0.999

Data are presented as mean ± standard deviation for continuous variables and as number (percentage) for categorical variables. *P* values were calculated using one-way ANOVA for continuous variables, Pearson's chi-squared test for sex, Kruskal–Wallis test for ASA physical status, and Fisher's exact test for medical history. ASA, American Society of Anesthesiologists; BMI, body mass index.

### 3.2 Emergence agitation

The overall incidences of EA differed significantly among the three groups (*χ*^2^ = 7.989, *P* = 0.018; Figure 2). *Post-hoc* pairwise comparisons with Bonferroni correction (*α*’ = 0.0167) revealed that Group E2 (3/31, 9.7%) had a significantly lower EA incidence compared with Group C (13/32, 40.6%; *χ*^2^ = 6.410, *P* = 0.011). This corresponds to an ARR of 30.9% (95% CI: 11.0% to 50.9%) and a NNT of 3.2 (95% CI: 2.0 to 9.1). Group E1 (8/32, 25.0%) showed a numerically lower but not statistically significant difference from Group C (*χ*^2^ = 1.134, *P* = 0.287). The comparison between Group E1 and Group E2 was not significant (*χ*^2^ = 1.612, *P* = 0.204). To evaluate the robustness of the primary finding, a sensitivity analysis was conducted in the FAS (mITT, *n* = 103). The incidence of EA in the mITT population was (14/34, 41.2%) in Group C, (9/35, 25.7%) in Group E1, and (3/34, 8.8%) in Group E2 (*χ*^2^ = 9.436, *P* = 0.009). Pairwise comparisons confirmed that Group E2 maintained a significantly lower risk of EA compared with Group C (*P* = 0.006), consistent with the per-protocol results. In exploratory subgroup analyses stratified by sex and age (Supplementary Table S1), the preventive effect of Group E2 was consistent across strata. No significant effect modification was detected by sex (*P* for interaction=0.812) or age (*P* for interactio*n* = 0.765).

**Figure 2:**
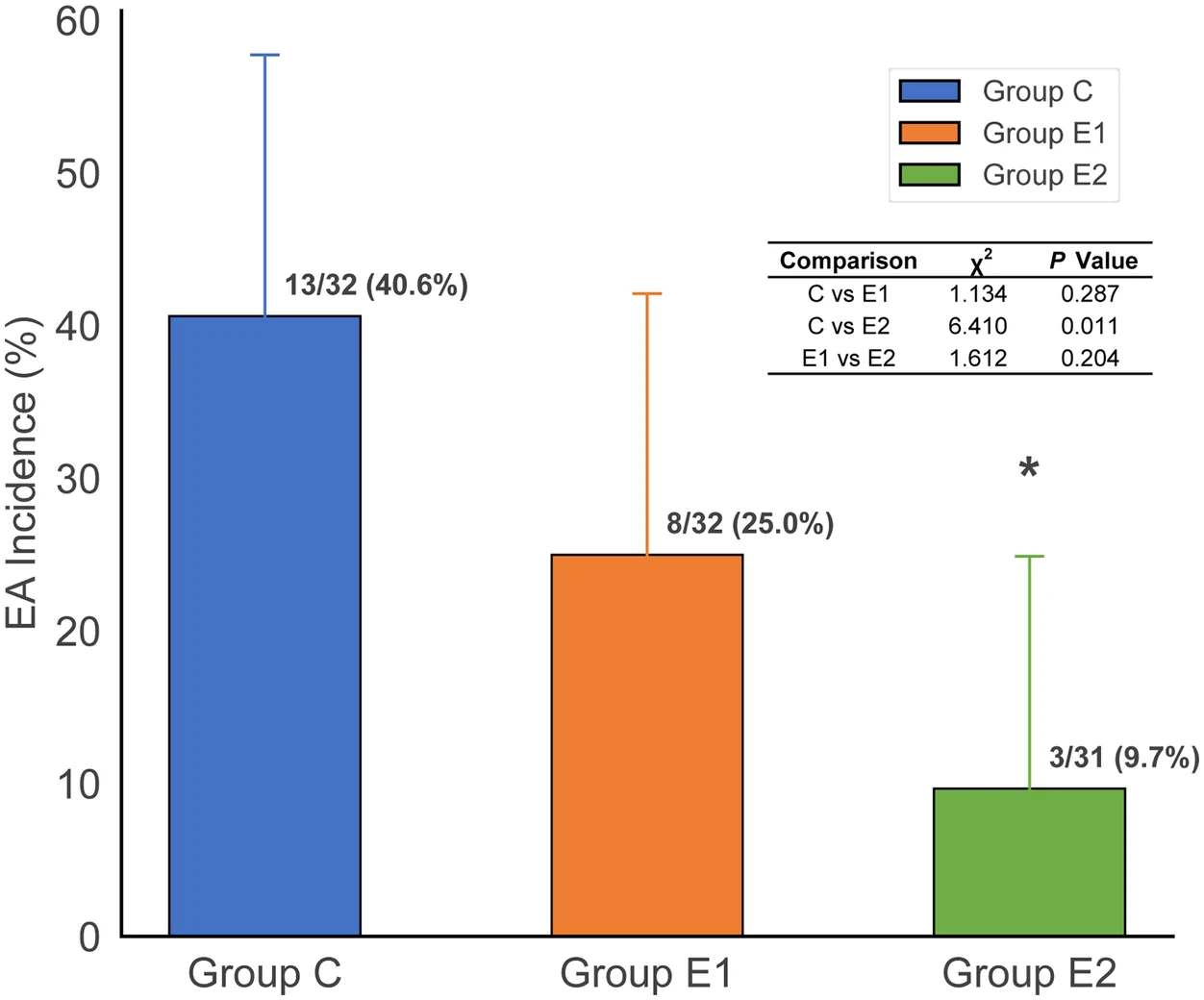
Incidence of emergence agitation among the three groups. The bar heights represent the percentage of patients who developed EA in each group. Error bars indicate the 95% confidence intervals of the proportions. Overall difference among the three parallel arms was assessed using the global omnibus chi-squared test (*χ*^2^ = 7.989, *P* = 0.018). *Post-hoc* pairwise comparisons were performed with Bonferroni correction (adjusted significance level *α*’ = 0.05/3 = 0.0167). The asterisk (*) indicates a statistically significant difference between Group C and Group E2 (*P* = 0.011 < *α*’). The inset table details the pairwise chi-squared statistics and unadjusted P values. EA, emergence agitation; Group C, control group; Group E1, esketamine at induction; Group E2, esketamine 20 min before the end of surgery.

SAS scores differed significantly among groups (Kruskal–Wallis H = 7.948, *P* = 0.019; Supplementary Figure S1). *Post-hoc* analysis with Bonferroni correction showed that SAS scores in Group E2 were significantly lower than in Group C (adjusted *P* = 0.016). No significant differences were found between Group C and Group E1 (adjusted *P* = 0.379) or between Group E1 and Group E2 (adjusted *P* = 0.569).

The exploratory multivariable logistic regression analysis incorporating the 24 total EA events across 95 patients revealed that Group E2 remained independently associated with a lower risk of EA compared with Group C (adjusted OR 0.117, 95% CI 0.027–0.511, *P* = 0.004; Table 2). Group E1 showed a numerically lower but not statistically significant odds ratio (adjusted OR 0.386, 95% CI 0.116–1.288, *P* = 0.122). No other covariates reached statistical significance (all *P* > 0.05).

**Table 2:** Multivariable logistic regression analysis of risk factors for emergence agitation.

Variable	*β*	SE	z Value	Adjusted OR	95% CI	*P* Value
Group (vs. Group C)						
Group E1	−0.952	0.615	−1.548	0.386	0.116–1.288	0.122
Group E2	−2.146	0.752	−2.854	0.117	0.027–0.511	0.004
Age (years)	−0.016	0.022	−0.705	0.984	0.943–1.028	0.481
Sex (Male vs. Female)	1.021	0.654	1.561	2.777	0.770–10.004	0.119
BMI (kg.m⁻^2^)	0.072	0.079	0.913	1.075	0.920–1.255	0.361
ASA status (Class II vs. I)	0.223	0.656	0.340	1.250	0.345–4.522	0.734
Duration of surgery (min)	0.003	0.009	0.283	1.003	0.985–1.020	0.777
Blood loss (ml)	−0.003	0.003	−1.008	0.997	0.992–1.002	0.314

This multivariable model is exploratory. The wide 95% confidence interval reflects the low events-per-variable ratio (total EA cases is 24/95) and should be interpreted with caution. β, regression coefficient; SE, standard error; OR, odds ratio; CI, confidence interval; BMI, body mass index; ASA, American Society of Anesthesiologists.

### 3.3 Postoperative pain

Rescue analgesic use in the PACU was comparable across groups (Group C: 8/32, 25.0%; Group E1: 6/32, 18.8%; Group E2: 3/31, 9.7%; *χ*^2^ = 2.540, *P* = 0.281; Supplementary Table S2). NRS pain scores at 2 h, 4 h, 6 h, and 24 h postoperatively did not differ significantly among the three groups (all *P* > 0.05; Table 3).

**Table 3:** Postoperative pain intensity scores and analgesic outcomes.

Parameter	Group C (*n* = 32)	Group E1 (*n* = 32)	Group E2 (*n* = 31)	*P* Value
Pain score at rest				
Postoperative 2 h	2 (0, 3)	1 (0, 2)	2 (0, 2)	0.785
Postoperative 4 h	2 (1, 2)	2 (1, 3)	2 (0, 2)	0.959
Postoperative 6 h	1 (1, 2)	2 (1, 2)	1 (1, 2)	0.832
Postoperative 24 h	1 (1, 2)	1 (1, 2)	1 (1, 2)	0.854
Rescue analgesia, n (%)	6 (18.8)	5 (15.6)	7 (22.6)	0.780

Data are presented as median (interquartile range, IQR) for pain scores and as number (percentage) for rescue analgesia. Pain scores were evaluated using the Numeric Rating Scale (NRS). *P* values were calculated using the Kruskal–Wallis test for pain scores and Pearson's chi-squared test for rescue analgesia. Rescue analgesia indicated postoperative rescue analgesia in the ward.

### 3.4 Hemodynamic parameters

Two-way mixed ANOVA showed a significant main effect of time on MAP (F = 81.846, *P* < 0.001, *η*^2^p = 0.471) and HR (F = 63.698, *P* < 0.001, *η*^2^*p* = 0.409; Supplementary Table S3, Supplementary Table S4, Supplementary Figure S2). However, there were no significant main effects of group on MAP (F = 1.556, *P* = 0.217) or HR (F = 1.464, *P* = 0.237), and no significant group-by-time interactions for MAP (F = 1.012, *P* = 0.439) or HR (F = 1.176, *P* = 0.289). These results indicate that all three groups exhibited similar hemodynamic patterns throughout the perioperative period.

BIS values showed a significant main effect of time (F = 944.373, *P* < 0.001, *η*^2^p = 0.911) and a nominally significant group-by-time interaction (F = 2.610, nominal *P* = 0.018, *η*^2^p = 0.054; Supplementary Table S5), while the main effect of group was not significant (F = 2.761, *P* = 0.069). Given the absence of multiplicity correction, this BIS interaction was strictly exploratory and hypothesis-generating. Importantly, this transient BIS elevation reflects the paradoxical electroencephalographic cortical activation induced by NMDA-receptor antagonists rather than inadequate anesthetic depth, with zero intraoperative awareness.

### 3.5 Intraoperative and postoperative data

Intraoperative blood loss differed at a nominal significance level among groups (Kruskal–Wallis H = 7.441, nominal *P* = 0.024; Supplementary Table S6), which was explicitly exploratory. Median blood loss was 20 ml in Group E1, compared with 50 ml in both Group C and Group E2. No significant intergroup differences were observed in surgery duration, anesthetic drug dosages (propofol, sufentanil, remifentanil), fluid administration, vasoactive drug consumption, extubation time, or PACU stay duration (all *P* > 0.05). Discharge time and time to nasal packing removal were similar across groups (*P* > 0.05; Supplementary Table S6).

### 3.6 Adverse events

The most common postoperative adverse event was dizziness, reported in 50.0% (16/32) of Group C, 37.5% (12/32) of Group E1, and 35.5% (11/31) of Group E2 (*P* = 0.444; Table 4). Nausea occurred in 6.3% (2/32), 0% (0/32), and 3.2% (1/31) of Groups C, E1, and E2, respectively (*P* = 0.541). Drowsiness was reported in 6.3% (2/32), 6.3% (2/32), and 9.7% (3/31) (*P* = 0.793). No vomiting occurred in any group. No psychiatric or neurological adverse events related to esketamine were observed. All adverse events were mild and self-limiting. None of the intergroup comparisons for adverse events reached statistical significance.

**Table 4:** Incidence of postoperative adverse events among groups.

Adverse Event	Group C n(%)	Group E1 n(%)	Group E2 n(%)	*P* Value
Nausea	2 (6.3)	0 (0.0)	1 (3.2)	0.541
Vomiting	0 (0.0)	0 (0.0)	0 (0.0)	—
Dizziness	16 (50.0)	12 (37.5)	11 (35.5)	0.444
Drowsiness	2 (6.3)	2 (6.3)	3 (9.7)	0.793
Sore throat	0 (0.0)	0 (0.0)	2 (6.5)	0.104
Urinary retention	2 (6.3)	0 (0.0)	0 (0.0)	0.326

Data are presented as number (percentage). *P* values were calculated using Pearson's chi-squared test or Fisher's exact test as appropriate based on expected cell counts. “-” denotes not calculated due to zero occurrences across all groups. Group C, control group; Group E1, esketamine at induction; Group E2, esketamine 20 min before the end of surgery.

## 4 Discussion

This randomized, double-blind, placebo-controlled trial demonstrated that the timing of subanesthetic esketamine administration critically influences its efficacy in preventing EA in patients undergoing nasal endoscopic surgery. Administration 20 min before the end of surgery (Group E2) reduced EA incidence from 40.6% to 9.7% (with ARR of 30.9% and NNT of 3.2), and significantly lowered SAS scores, while administration during induction (Group E1) did not achieve statistical significance compared with the control group. Crucially, these preventive benefits were achieved without causing hemodynamic instability, prolonging extubation or PACU recovery times, or inducing psychotomimetic adverse events.

The timing-dependent efficacy observed in our study is strongly supported by the pharmacokinetic (PK) and pharmacodynamic (PD) profiles of esketamine. Extubation is one of the most potent stimuli during emergence, triggering marked sympathetic activation, hypertension, and tachycardia. For nasal surgery patients, nasal packing adds sustained discomfort and respiratory distress, further amplifying the stress response, drastically amplifying the risk of EA (20–22).

In Group E2, administering esketamine 20 min prior to surgical completion ensured that its peak plasma concentration and central sedative/anti-hyperalgesic effects precisely coincided with the vulnerable window of extubation and immediate postoperative nasal discomfort. This optimal concentration gradient suppressed central hyperexcitability and facilitated a calm emergence trajectory. This is consistent with previous studies demonstrating that ketamine administration 10-20 min before surgery end provides optimal stress suppression (19, 23). Conversely, in Group E1, the drug was administered at induction (approximately 60–90 min prior to extubation). By the time of emergence, the peak pharmacodynamic effect had likely waned, with plasma concentrations falling below the threshold needed to fully suppress the stress response. The numerically lower EA rate in Group E1 (25.0% vs. 40.6% in Group C) suggests a residual protective effect, but one that was insufficient to reach statistical significance. This observation highlights that esketamine's preventive effect depends not merely on whether it is administered, but on when it is administered relative to the emergence period.

Despite the known analgesic properties of esketamine at subanesthetic doses, we observed no significant differences in NRS pain scores at any postoperative time point. This null finding aligns with some previous studies. Sun et al.'s meta-analysis of breast cancer surgery patients found no significant reduction in pain scores up to 3 months postoperatively with perioperative ketamine or esketamine (24). Several factors may explain this observation. First, pain associated with nasal endoscopic surgery is predominantly caused by the compressive sensation, mucosal stretch, obligatory mouth-breathing suffocation and foreign body feeling caused by bilateral nasal packing rather than deep tissue injury (25). This discomfort, while distressing, may not be adequately captured by the NRS, which is designed specifically for pain assessment. Second, the analgesic effect of a single bolus dose may not persist throughout the entire nasal packing period (48-72 h). Third, the routine administration of flurbiprofen axetil 50 mg for preventive analgesia in all groups may have contributed to a floor effect, limiting the ability to detect between-group differences. Future studies should consider employing assessment tools that separately capture pain and discomfort related to nasal packing, and explore multimodal analgesic regimens that combine esketamine with non-steroidal anti-inflammatory drugs or local anesthetics (26).

Cardiovascular stability is an essential prerequisite for perioperative neuroprotection. Although racemic ketamine is classically recognized for sympathomimetic stimulation, subanesthetic esketamine (0.25 mg).kg^−1^) in our cohort did not provoke clinically relevant increases in MAP or HR relative to placebo. Repeated-measures ANOVA confirmed stable hemodynamic trajectories across all groups, reflecting balanced anesthesia maintained by target-controlled infusion of propofol and remifentanil.

Notably, in Group E2, a transient elevation in BIS values was observed near the end of surgery. Rather than indicating lightened anesthesia or risk of intraoperative awareness, this phenomenon represents a well-documented electroencephalographic (EEG) signature of NMDA receptor antagonists, which induce high-frequency frontoparietal *β*/*γ* oscillations while preserving adequate depth of unconsciousness (27). Importantly, no patient experienced intraoperative awareness or delayed emergence.

The overall safety profile was excellent. The incidence of common minor adverse events was low and statistically indistinguishable across groups. Most importantly, zero patients reported hallucinations, nightmares, or dissociative symptoms, reinforcing the established clinical safety margin of subanesthetic esketamine (≤0.3 mg.kg^−1^) over higher anesthetic doses or racemic ketamine (12, 13, 28).

An unexpected finding was the significantly lower intraoperative blood loss in Group E1 (median 20 ml) compared with Group C and Group E2. We explicitly emphasize that intraoperative blood loss was an unplanned, exploratory *post-hoc* endpoint. We speculate that administering esketamine during induction may have attenuated the profound vasodilation and transient hypotension often induced by propofol and opioids, maintaining optimal microvascular perfusion pressure and capillary tone in the nasal mucosa during early surgical dissection. No causal conclusions should be drawn from this incidental finding. Crucially, this single-center observation cannot guide clinical practice, which requires external validation. As blood loss was not a pre-specified primary outcome, this finding should be interpreted with caution and requires validation in future prospective studies with planned hemodynamic stratification and coagulation function assessment (29).

This study has several limitations. First, the sample size, while adequate for the primary outcome, may have been insufficient to detect small-to-moderate differences in secondary outcomes such as pain scores and subgroup analyses. Therefore, significant secondary findings should be interpreted with caution and not conflated with definitive therapeutic equivalence. Second, this was a single-center trial conducted exclusively in adult nasal endoscopic surgery. Caution is required before generalizing these findings to pediatric populations or other surgical specialties. Third, although Riker SAS is a validated and widely used tool for EA assessment, it inherently involves subjective judgment, which may introduce inter-rater variability despite standardized training and blinding. Fourth, we evaluated only a single subanesthetic dose (0.25 mg.kg^−1^) across two discrete time points; whether alternative doses (e.g., 0.15 or 0.3 mg.kg^−1^) or continuous infusions offer superior efficacy, and the precise dose-timing interaction, remains to be established. Fifthly, postoperative pain assessment relied exclusively on the unidimensional NRS, which captures somatic nociceptive intensity but fails to adequately quantify the subjective distress, foreign-body sensation, and suffocation-like discomfort induced by bilateral nasal packing. Finally, the Fragility Index of our primary comparison (Group C vs. Group E2) is 1. As described by Walsh et al. (30), this indicates that reversing the outcome of just a single patient in the intervention group would alter the statistical significance based on our strict Bonferroni-corrected threshold (*α*'= 0.0167). This fragility highlights that while our findings are statistically significant, they are sensitive to small event counts, underscoring the necessity for larger, multicenter trials to confirm the robustness of this intervention.

## 5 Conclusions

In adult patients undergoing nasal endoscopic surgery under general anesthesia, intravenous administration of a subanesthetic dose of esketamine (0.25 mg.kg⁻^1^) 20 min before the end of surgery significantly reduces the incidence and severity of emergence agitation compared to placebo. While this appears to be a promising and safe prophylactic strategy that does not delay recovery, the fragility of the primary outcome and the single-center design limit immediate widespread clinical adoption. Large-scale, multicenter trials are warranted to externally validate these findings and optimize dose-timing interactions before widespread routine clinical adoption.

## Data Availability

The raw data supporting the conclusions of this article will be made available by the authors, without undue reservation.
