# Supplementary material for: Impact of administration timing of subanesthetic doses of esketamine on emergence agitation in patients undergoing nasal endoscopic surgery: a randomized controlled trial
Source: Front Med (Lausanne). 2026 Oct 28;13:1978040. doi: 10.3389/fmed.2026.1978040 (PMC13751460; doi:10.3389/fmed.2026.1978040)
Supplement: Supplementary file 1 [file Supplementaryfile1.docx]

Supplementary Material

**Figure S1. Distribution of peak Sedation-Agitation Scale scores in the post-anesthesia care unit.**

Notes: The violin plots illustrate the probability density of the SAS scores for each group, with wider sections indicating a higher frequency of patients at that specific score. The internal white circles represent individual patient data points, and the central horizontal black lines denote the median values. The vertical lines indicate the range of the scores. Overall significance was assessed using the Kruskal-Wallis test, and the inset table details the post-hoc pairwise comparisons performed using the Mann-Whitney U test. Adjusted P values were calculated using the Bonferroni correction for multiple comparisons. A statistically significant reduction in SAS scores is observed in Group E2 compared with Group C (Adjusted *P* = 0.016).

Abbreviations: SAS, Sedation-Agitation Scale; Group C, control group; Group E1, esketamine at induction; Group E2, esketamine 20 min before the end of surgery.

**Figure S2. Perioperative hemodynamic and depth of anesthesia parameters across the three groups.**

Notes: Changes in (A) mean arterial pressure (MAP), (B) heart rate (HR), and (C) bispectral index (BIS) are presented. Data are expressed as mean ± standard deviation (SD). Time points are defined as follows: T0, pre-induction; T1, immediately after intubation; T2, 10 min after intubation; T3, end of surgery; T4, PACU entry; T5, extubation; T6, 10 min after extubation; T7, PACU discharge. BIS values were monitored and recorded from T0 to T3. Data were analyzed using a two-way mixed ANOVA. For MAP and HR, there were significant main effects of time (P < 0.001), but no significant main effects of group or group-by-time interactions (P > 0.05). For BIS, there was a significant main effect of time (P < 0.001) and a significant group-by-time interaction (P = 0.018).

Abbreviations: MAP, mean arterial pressure; HR, heart rate; BIS, bispectral index; PACU, post-anesthesia care unit; bpm, beats per minute; Group C, control group; Group E1, esketamine at induction; Group E2, esketamine 20 min before the end of surgery.
