# Supplementary material for: Impact of administration timing of subanesthetic doses of esketamine on emergence agitation in patients undergoing nasal endoscopic surgery: a randomized controlled trial
Source: Front Med (Lausanne). 2026 Oct 28;13:1978040. doi: 10.3389/fmed.2026.1978040 (PMC13751460; doi:10.3389/fmed.2026.1978040)
Supplement: Supplementary file 2 [file Table1.docx]

Supplementary Material

**Table S1. Stratified Analysis of Emergence Agitation by Sex and Age.**

| Subgroup | Group E1  (n=32) | Group E2  (n=31) | Group C  (n=32) | Overall  P value | P for  Interaction | E1 vs C  (*P*) | E2 vs C  (*P*) |
| --- | --- | --- | --- | --- | --- | --- | --- |
| Sex |  |  |  |  | 0.812 |  |  |
| Male (n=69) | 6/21 (28.6%) | 3/25 (12.0%) | 11/23 (47.8%) | 0.024 |  | 0.228 | 0.010 |
| Female (n=26) | 2/11 (18.2%) | 0/6 (0.0%) | 2/9 (22.2%) | 0.642 |  | 1.000 | 0.486 |
| Age |  |  |  |  | 0.765 |  |  |
| ≤42 yr (n=49) | 4/16 (25.0%) | 2/16 (12.5%) | 7/17 (41.2%) | 0.173 |  | 0.465 | 0.118 |
| >42 yr (n=46) | 4/16 (25.0%) | 1/15 (6.7%) | 6/15 (40.0%) | 0.109 |  | 0.458 | 0.080 |

Notes: Data are presented as event count / total number (percentage), n/N (%). *P* values were calculated by Fisher’s exact test. P for interaction was evaluated using logistic regression models including the treatment-by-subgroup interaction term. Subgroup analyses are exploratory and were not pre-powered to detect subtle interactions, particularly within the female subset (n = 26).
