# Supplementary material for: Impact of administration timing of subanesthetic doses of esketamine on emergence agitation in patients undergoing nasal endoscopic surgery: a randomized controlled trial
Source: Front Med (Lausanne). 2026 Oct 28;13:1978040. doi: 10.3389/fmed.2026.1978040 (PMC13751460; doi:10.3389/fmed.2026.1978040)
Supplement: Supplementary file 3 [file Table2.docx]

Supplementary Material

**Table S2: Rescue medication administration in the PACU**

| **Variable** | **Group C  (n = 32)** | **Group E1  (n = 32)** | **Group E2  (n = 31)** | **Statistic** | ***P* Value** |
| --- | --- | --- | --- | --- | --- |
| Rescue drug use, n(%) | 8 (25.0) | 6 (18.8) | 3 (9.7) | χ²=2.540 | 0.281 |
| Rescue propofol (mg) | 0 (0, 13.0) | 0 (0, 10.0) | 0 (0, 7.0) | H=3.252 | 0.197 |
| Rescue sufentanil (µg) | 0 (0, 5.0) | 0 (0, 5.0) | 0 (0, 5.0) | H=0.493 | 0.782 |

Notes: Data are presented as number (percentage) for categorical variables and median (interquartile range, IQR) for continuous variables. P values were calculated using Pearson's chi-squared test (χ2) for the incidence of rescue drug use, and the Kruskal-Wallis test (H) for the dosages of propofol and sufentanil. Abbreviations: PACU, post-anesthesia care unit.
