# Supplementary material for: Impact of administration timing of subanesthetic doses of esketamine on emergence agitation in patients undergoing nasal endoscopic surgery: a randomized controlled trial
Source: Front Med (Lausanne). 2026 Oct 28;13:1978040. doi: 10.3389/fmed.2026.1978040 (PMC13751460; doi:10.3389/fmed.2026.1978040)
Supplement: Supplementary file 4 [file Table3.docx]

Supplementary Material

**Table S3. Serial Changes in Mean Arterial Pressure (MAP) Over Time Among Groups**

| **Time Point** | **Group C  (n = 32)** | **Group E1  (n = 32)** | **Group E2  (n = 31)** |
| --- | --- | --- | --- |
| T0 (Pre-induction) | 92.41 ± 9.81 | 100.06 ± 11.68 | 96.87 ± 9.98 |
| T1 (Post-intubation) | 83.41 ± 12.07 | 85.81 ± 16.37 | 87.52 ± 11.20 |
| T2 (10 min post-intubation) | 74.66 ± 10.91 | 77.25 ± 11.69 | 78.35 ± 10.91 |
| T3 (End of surgery) | 81.09 ± 8.27 | 80.56 ± 10.80 | 79.00 ± 8.93 |
| T4 (PACU entry) | 92.56 ± 11.82 | 93.62 ± 17.80 | 91.48 ± 15.16 |
| T5 (Extubation) | 99.62 ± 12.06 | 101.66 ± 15.41 | 103.61 ± 11.52 |
| T6 (10 min post-extubation) | 93.69 ± 9.23 | 99.75 ± 12.50 | 98.35 ± 12.44 |
| T7 (PACU discharge) | 94.78 ± 8.26 | 99.34 ± 13.50 | 99.35 ± 12.85 |

Notes: Data are presented as mean ± standard deviation in mmHg. Two-way repeated-measures ANOVA with Greenhouse-Geisser correction revealed: Group effect: F = 1.556, P = 0.217, partial η² = 0.033; Time effect: F = 81.846, P < 0.001, partial η² = 0.471; Group × Time interaction: F = 1.012, P = 0.439, partial η² = 0.022. Abbreviations: MAP, mean arterial pressure; PACU, post-anesthesia care unit; ANOVA, analysis of variance.
