# Supplementary material for: Impact of administration timing of subanesthetic doses of esketamine on emergence agitation in patients undergoing nasal endoscopic surgery: a randomized controlled trial
Source: Front Med (Lausanne). 2026 Oct 28;13:1978040. doi: 10.3389/fmed.2026.1978040 (PMC13751460; doi:10.3389/fmed.2026.1978040)
Supplement: Supplementary file 5 [file Table4.docx]

Supplementary Material

**Table S4. Serial Changes in Heart Rate (HR) Over Time Among Groups**

| **Time Point** | **Group C  (n = 32)** | **Group E1  (n = 32)** | **Group E2  (n = 31)** |
| --- | --- | --- | --- |
| T0 (Pre-induction) | 75.41 ± 11.18 | 82.03 ± 13.28 | 81.55 ± 16.72 |
| T1 (Post-intubation) | 72.50 ± 13.10 | 73.38 ± 14.94 | 75.03 ± 13.94 |
| T2 (10 min post-intubation) | 58.91 ± 11.11 | 59.97 ± 9.97 | 60.19 ± 11.48 |
| T3 (End of surgery) | 64.97 ± 9.83 | 63.72 ± 11.03 | 66.52 ± 10.37 |
| T4 (PACU entry) | 73.00 ± 13.83 | 74.09 ± 16.49 | 73.42 ± 14.80 |
| T5 (Extubation) | 82.09 ± 14.04 | 80.75 ± 14.13 | 89.13 ± 17.40 |
| T6 (10 min post-extubation) | 77.28 ± 10.26 | 79.34 ± 11.26 | 83.81 ± 15.92 |
| T7 (PACU discharge) | 76.16 ± 9.06 | 78.53 ± 12.01 | 81.68 ± 13.03 |

Notes: Data are presented as mean ± standard deviation in beats per minute (bpm). Two-way repeated-measures ANOVA with Greenhouse-Geisser correction revealed: Group effect: F = 1.464, P = 0.237, partial η² = 0.031; Time effect: F = 63.698, P < 0.001, partial η² = 0.409; Group × Time interaction: F = 1.176, P = 0.289, partial η² = 0.025. Abbreviations: HR, heart rate; PACU, post-anesthesia care unit; ANOVA, analysis of variance.
