# Supplementary material for: Impact of administration timing of subanesthetic doses of esketamine on emergence agitation in patients undergoing nasal endoscopic surgery: a randomized controlled trial
Source: Front Med (Lausanne). 2026 Oct 28;13:1978040. doi: 10.3389/fmed.2026.1978040 (PMC13751460; doi:10.3389/fmed.2026.1978040)
Supplement: Supplementary file 6 [file Table5.docx]

Supplementary Material

**Table S5: Serial Changes in Bispectral Index (BIS) Values Over Time Among Groups**

| **Time Point** | **Group C  (n = 32)** | **Group E1  (n = 32)** | **Group E2  (n = 31)** |
| --- | --- | --- | --- |
| T0 (Pre-induction) | 93.28 ± 3.14 | 94.00 ± 2.83 | 94.16 ± 2.65 |
| T1 (Post-intubation) | 49.31 ± 11.17 | 52.81 ± 10.42 | 51.48 ± 7.13 |
| T2 (10 min post-intubation) | 48.44 ± 6.52 | 51.53 ± 6.42 | 49.58 ± 6.14 |
| T3 (End of surgery) | 53.03 ± 6.23 | 53.12 ± 8.90 | 59.16 ± 9.69 |

Notes: Data were presented as mean ± standard deviation. Two-way repeated-measures ANOVA revealed: Group effect: F = 2.761, P = 0.069, partial η² = 0.057; Time effect: F = 944.373, P < 0.001, partial η² = 0.911; Group × Time interaction: F = 2.610, P = 0.018, partial η² = 0.054. The nominally significant interaction was unadjusted for multiplicity and should be regarded as exploratory.
