# Supplementary material for: Impact of administration timing of subanesthetic doses of esketamine on emergence agitation in patients undergoing nasal endoscopic surgery: a randomized controlled trial
Source: Front Med (Lausanne). 2026 Oct 28;13:1978040. doi: 10.3389/fmed.2026.1978040 (PMC13751460; doi:10.3389/fmed.2026.1978040)
Supplement: Supplementary file 7 [file Table6.docx]

Supplementary Material

**Table S6: Intraoperative and Postoperative Characteristics**

| **Variable** | **Group C  (n = 32)** | **Group E1  (n = 32)** | **Group E2  (n = 31)** | ***P* Value** |
| --- | --- | --- | --- | --- |
| Duration of surgery (min) | 85.0 (68.0, 97.8) | 73.0 (59.0, 111.2) | 70.0 (57.5, 86.0) | 0.417 |
| Intraoperative anesthetics |  |  |  |  |
| Propofol (mg) | 587.0 (472.5, 742.2) | 653.0 (442.0, 769.3) | 528.4 (446.5, 699.4) | 0.567 |
| Sufentanil (µg) | 26.4 (22.8, 28.7) | 25.8 (23.2, 29.2) | 25.6 (23.0, 29.4) | 0.999 |
| Remifentanil (µg) | 893.0 (702.5, 956.8) | 904.9 (736.7, 1265.5) | 804.0 (690.9, 1103.4) | 0.456 |
| Intraoperative blood loss (mL) | 50.0 (20.0, 200.0)^a^ | 20.0 (10.0, 50.0)^b^ | 50.0 (20.0, 100.0)^ab^ | 0.024 |
| Time to extubation (min) | 15.5 (9.8, 22.2) | 19.0 (12.8, 30.0) | 19.0 (14.0, 24.5) | 0.101 |
| Length of stay in PACU (min) | 40.0 (30.0, 50.0) | 42.0 (35.8, 49.0) | 40.0 (36.5, 54.0) | 0.314 |
| Duration of hospital stay (days) | 3.5 (3.0, 5.0) | 4.0 (3.0, 4.0) | 3.0 (3.0, 4.0) | 0.497 |
| Time to nasal pack removal (days) | 3.0 (3.0, 3.2) | 3.0 (2.0, 3.0) | 3.0 (2.0, 3.0) | 0.384 |

Notes: Data are presented as median (interquartile range). *P* values were calculated using the Kruskal-Wallis test. Different superscript letters within a row indicate a statistically significant difference (*P* < 0.05) between groups based on post-hoc Dunn’s pairwise comparison test. Secondary outcomes were not adjusted for multiple comparisons, so the nominally significant difference in blood loss is exploratory and hypothesis-generating. Abbreviations: PACU, post-anesthesia care unit; Group C, control group; Group E1, esketamine at induction; Group E2, esketamine 20 min before the end of surgery.
